# Needs and Demands for eHealth Pain Management Interventions in Chronic Pain Patients

**DOI:** 10.3390/jpm13040675

**Published:** 2023-04-17

**Authors:** Paula Stoppok, Anna-Lena Frewer, Adam Schweda, Sheila Geiger, Eva-Maria Skoda, Diana Müßgens, Ulrike Bingel, Martin Teufel, Alexander Bäuerle

**Affiliations:** 1 Clinic for Psychosomatic Medicine and Psychotherapy, University of Duisburg-Essen, LVR-University Hospital Essen, 45147 Essen, Germany; paula.stoppok@web.de (P.S.);; 2 Center for Translational Neuro- and Behavioral Sciences (C-TNBS), University of Duisburg-Essen, 45147 Essen, Germany; 3 Department of Neurology, Center for Translational Neuro- and Behavioral Sciences, University Hospital Essen, 45147 Essen, Germany

**Keywords:** eHealth, telehealth, chronic pain, pain treatment, tailored interventions

## Abstract

Although chronic pain is a global health problem, the current care situation is often inadequate. eHealth offers many advantages as an additional option for treating chronic pain. Yet, an intervention’s efficacy can only be fully exhausted if patients intend to use it. This study aims to identify the needs and demands of patients with chronic pain regarding intervention concepts and frameworks to develop specifically tailored eHealth pain management interventions. A cross-sectional study was conducted, including 338 individuals with chronic pain. Within the cohort, a distinction between a high- and a low-burden group was made. Respondents generally preferred a permanently accompanying mobile app, but the preferred content varied with group. According to the majority, interventions should be made available on smartphones, offer sessions once per week with a duration from 10 to 30 min, and be recommended by experts. These results can provide the basis for future eHealth pain management interventions tailored to the patients’ needs and demands.

## 1. Introduction

Chronic pain is recognized as a global health problem, but the care situation for patients with chronic pain is inadequate. According to Breivik and colleagues, 40 percent of patients with chronic pain are unsatisfied with their pain treatment and report having no adequate management for their pain [1]. During the COVID-19 pandemic, the care situation for patients with chronic pain was aggravated. On the one hand, the COVID-19 pandemic was suspected to increase the risks of developing chronic pain [2,3]. On the other hand, access to many pain management facilities was restricted by social distancing and COVID-19-related policies [2]. A study conducting interviews with chronic pain patients in Germany found that about one third of patients experienced a worsening of their chronic pain due to the pandemic [4]. To provide options for treating patients with chronic pain while adhering to social distancing, eHealth pain management interventions can offer a good supplement to face-to-face therapy [2,3,5]. eHealth provides the advantages of cost-effectiveness, ease of implementation, and location flexibility [6,7], and can thus offer anonymous support at a low threshold [7,8].

In addition to the assumed benefits, evidence is needed to meaningfully integrate eHealth into medical care [9,10]. A recent meta-analysis that investigated the efficacy of eHealth pain management interventions suggested that app-based treatment can be helpful in reducing pain, especially in the long term [7]. Comparable beneficial effects were reported in a further review [11]. Yet, the effect of eHealth interventions strongly depends on patients’ intentions to use them [12,13]. Furthermore, the quality of available pain apps could be improved by more user engagement in the development of the apps [14,15].

To date, eHealth interventions are often developed with little involvement of the future user [15,16]. This partly explains the high dropout rates regarding the use of eHealth [17]. High dropout rates are a common problem in eHealth studies [17]. According to Eysenbach [17], trials on the efficacy of eHealth interventions start with 100 percent of participants intending to use these interventions. Yet, the intention to use drops quickly, resulting in non-usage and high dropout rates. In a review of eHealth interventions for people affected by chronic pain, the average dropout rate was 26.6 percent [8].

To prevent high dropout rates, not only in the trials but also in real-world settings, the patients’ needs and demands should be addressed in developing eHealth interventions to increase actual use and, subsequently, the intervention’s effectiveness. A 2020 study in Norway has already applied a user-centered approach to the development of an eHealth pain management intervention. Various stakeholders, including patients, were involved in the development process [15]. The aim of the present study is now to determine the needs and demands for eHealth pain management interventions, including factors regarding content and design, by surveying a larger cohort of patients with chronic pain in Germany. It further aims to identify differences in the needs and demands depending on the self-perceived burden of chronic pain. Further, since data collection took place during the COVID-19 pandemic, an additional aim was to characterize the development of chronic pain during the pandemic, together with possible changes in medical care.

## 2. Materials and Methods

The participants were recruited via flyers and posters. These were distributed in multiple hospitals in Essen, Germany, at physician and physiotherapist offices throughout Germany, as well as via social media (i.e., pain-related support groups, German speaking). There was no financial compensation for participating in the study. The survey was conducted anonymously. The study was approved by the Ethics Committee of the Medical Faculty of the University Duisburg-Essen (19-89-47-BO).

Patients had to meet several inclusion criteria to participate in this cross-sectional study. First, they had to be at least 18 years old (of legal age). As a condition of participation, participants had to meet the diagnostic criteria for chronic pain according to the ICD-11 diagnosis code MG30 (chronic primary pain) [18]. Accordingly, patients had to confirm that they were currently still suffering from pain. Furthermore, speaking German, having internet access, and giving electronic informed consent at the beginning of the study were obligatory.

Before the statistical data analysis, participants who did not meet the inclusion criteria or who did not complete the survey were excluded. A total of 525 people took part in the study. Furthermore, 342 participants completed the assessment, of which 340 met the inclusion criteria. Two participants were subsequently excluded due to missing values, resulting in a total sample size of 338 (64.38%).

The survey included a variety of different scales. First, participants provided sociodemographic data on their gender, age, relationship status, educational qualifications, employment, and place of residence. Subsequently, medical data regarding chronic pain diagnosis criteria [18], prior treatment, and detailed symptom descriptions were assessed.

The following assessment instruments were used.

Pain Disability Index (PDI) [19]: The self-perceived level of disability caused by pain was assessed with the PDI, which consists of seven items (e.g., “How much does your pain prevent you from leading a normal life?”). Participants indicate their disability due to pain on a 6-point Likert scale (1 = never to 6 = always). The pain disability index is represented using a sum score (7–42), with a higher value meaning a greater severity. Cronbach’s α in this study was 0.91, indicating excellent internal consistency [20].

Veterans RAND 12-Item Health Survey (VR-12) [21,22,23,24]: As part of the German pain questionnaire, the VR-12 proved to be an adequate assessment instrument for recording the self-rated HRQoL (Health-Related Quality of Life) in pain patients [23]. It consists of twelve items (e.g., “I accomplished less than I wanted to.”). Participants can answer on a 4-point Likert scale (ranging from 0 = not at all to 3 = nearly every day). According to Hüppe et al. [23], the scale results in a physical (PCS) and a mental component score (MCS). High values correspond to a high level of physical and mental health. In detail, scores of ≤40 indicate poor HRQoL. Cronbach’s α in this study was 0.91, indicating excellent internal consistency [20].

Questionnaire for the assessment of pain-specific self-efficacy (abbreviation of German scale: FESS) [25]: As an adaption of the Pain Self-Efficacy Questionnaire (PSEQ) [26], the FESS comprises ten items. They generally ask to what extent subjects are convinced that they can carry out certain activities “despite the pain” (e.g., “I can enjoy things despite the pain.”). Answers are given on a 6-point Likert scale (from “completely convinced” to “not at all convinced”). The pain-specific self-efficacy is operationalized by a sum score (range = 10–60), with a higher value representing a greater degree of pain-specific self-efficacy. Cronbach’s α in this study was 0.93, which indicates excellent internal consistency [20].

Several self-generated items that assessed the participants’ needs and demands regarding an eHealth intervention were included, see Appendix B, Table A1. These items had different options to answer (e.g., dichotomous, Likert, single and multiple choice). Internet use, attitudes toward and experiences with online interventions.

Six self-generated items assessed the impact of COVID-19 on chronic pain. The following topics were included: cancellation of treatment appointments, change in pain symptoms, and potential reasons for it. Participants had to (dis)agree to statements such as “I was in more pain” via dichotomous answer options (yes/no) or ticking the applicable statement.

The statistical analyses were performed using RStudio 4.2.1 [27]. Before performing any statistical test, the relevant assumptions were tested. First, internal consistencies, descriptive statistics, and sum/mean scores for the different psychometric questionnaires were calculated (see Section 3.1.).

To investigate needs and demands of patients with chronic pain, associated variables (i.e., internet use, previous experiences with eHealth, needs and demands regarding eHealth, and the everyday relevance of eHealth) were analyzed descriptively (including absolute and percentual frequencies).

Subsequently, potential interindividual differences in the needs and demands regarding eHealth pain management of those affected by chronic pain were examined based on chronic pain psychopathological conditions. A cluster analysis of the three core psychometric characteristics (i.e., VR-12, FESS, PDI) was performed to explore potential subgroups within the sample. Since the data set was not subjected to any outlier correction and the *k*-medoids method algorithm is more robust to outliers than the k-means algorithm [28], this method was preferred. The suitability for clustering was checked for the total scores for HRQoL, pain-specific self-efficacy, and perceived pain disability using the Hopkins’ *H*-statistic, which evaluates the data’s clustering tendency. The optimal number of clusters was identified with the “*n*-clusters” command from the parameter package [29]. A two-cluster *k*-medoids method cluster analysis was then computed. The overall model performance was assessed using simple *R*^2^-statistics. Classifiability was further checked using linear discrimination analyses. K-means yielded very similar results.

Chi-Square tests, Fisher’s exact tests (for categorial data), and Wilcoxon signed-rank tests (for continuous data) were used to examine potential differences in the needs and demands based on the participants’ cluster (α = 0.05).

## 3. Results

### 3.1. Sociodemographic, Medical, and Psychometric Data

The average age of the participants was 45.56 years (*SD* = 10.73, range = 18–69 years). Most (*n* = 282, 83.43%) reported suffering from pain for a period of more than two years. Pain frequency was reported as permanent by 158 participants (46.75%) and as daily by 109 participants (32.25%). Furthermore, 237 of the 338 participants (70.12%) reported having started therapy, including physiotherapy, medication, psychotherapy, etc., more than two years ago. Further sociodemographic and psychometric data of the sample are summarized in Table 1.

### 3.2. Needs and Demands of Chronic Pain Patients

#### 3.2.1. Internet Usage

**Figure A1:**
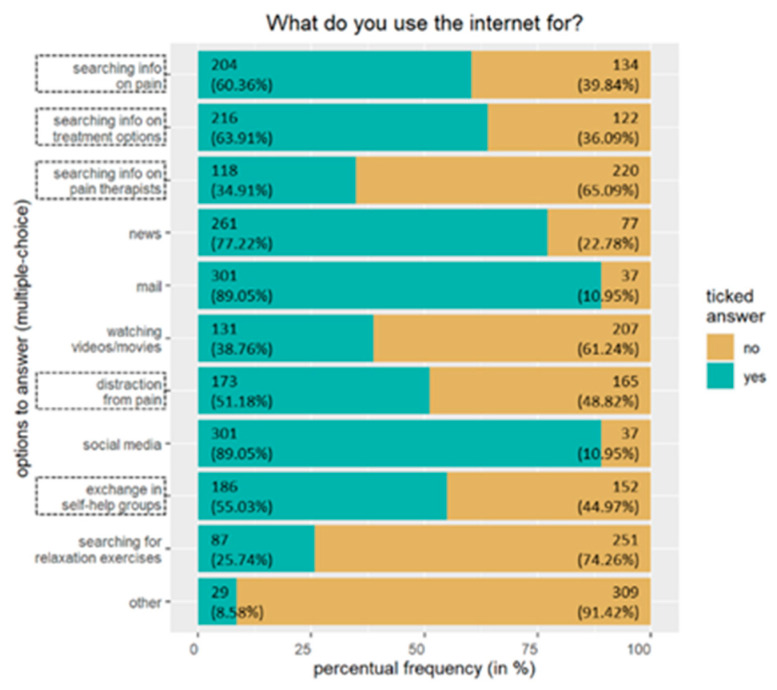
*n* = 338. The bars show absolute frequency (percentual frequency). Shown above are the participants’ responses regarding the thematic aim of their internet usage. Pain-related content is highlighted with a dashed box.

**Figure A2:**
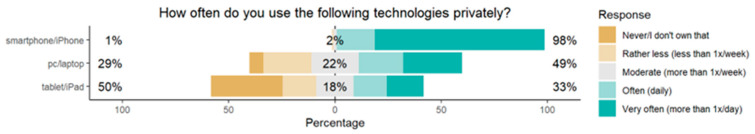
*n* = 329. Nine subjects were excluded since they did not answer the question fully. Shown above are the participants’ responses regarding privately, i.e., separate from work used technologies. Deviations in the direction of approval and rejection are shown on the right or left in percentual frequencies.

A total of 335 participants confirmed having private internet access (99.11%). More than half of the participants (59.76%) indicated accessing the internet for one to three hours daily. On average, more than half of the participants stated using the internet in the context of chronic pain (60.36%); see Figure A1. Nearly all participants reported using their smartphone at least once per day; see Figure A2.

#### 3.2.2. eHealth: Prior Experiences

**Figure A3:**
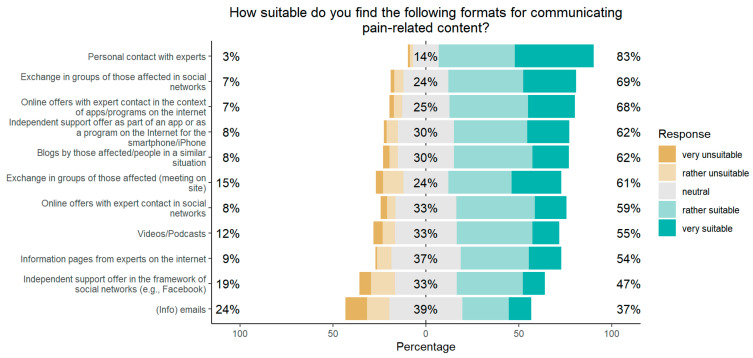
*n* = 327. Eleven subjects were excluded since they did not answer the question fully. Shown above are the participants’ responses regarding formats’ suitability for communicating pain-related content. Deviations in the direction of approval and rejection are shown on the right or left in percentual frequencies.

Overall, participants rated most formats suitable for communicating content relevant to pain; see Figure A3. The majority of participants preferred personal contact with experts. On the other hand, (info) emails were the least preferred format for communicating pain-related content.

In total, 227 participants (67.16%) stated having no prior experience with online pain management services and were not aware of their existence. Although 112 participants reported being aware of such online offers, only 39 of those affirmed having used such an offer in the past, and 32 participants indicated which online offers for pain management they had already used.

#### 3.2.3. eHealth: Needs and Demands

**Figure A4:**
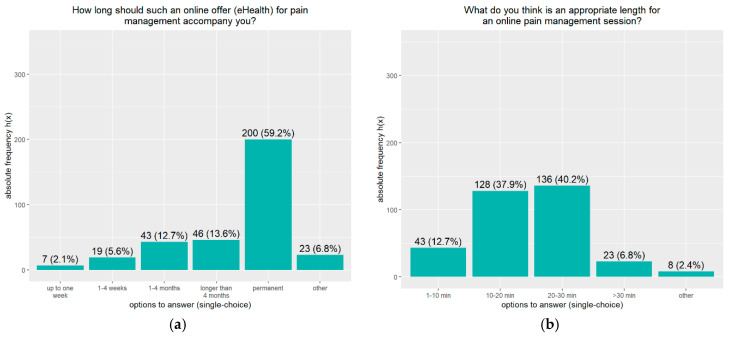
*n* = 338. The bars show absolute frequency (percentual frequency). Shown above are the participants’ responses regarding (**a**) the duration of an online offer for pain management; (**b**) one session’s length of an online offer for pain management.

Most participants (59.17%) would like to permanently use online pain management offers; see Figure A4a. Still, 13.61 percent (*n* = 46) desired an offer for at least four months and 12.72 percent (*n* = 43) for at least one to four months. Only 2.07 percent (*n* = 7) would prefer an offer for only one week. Regarding the online offers’ frequency, about two-thirds reported preferring weekly sessions. In total, 264 participants (78.11%) preferred each online pain management session to be 10–30 min long; see Figure A4b. In total, 12.72 percent (*n* = 43) wished for a shorter offer of one to ten minutes. Only 6.80 percent (*n* = 23) preferred an extended offer of more than 30 min.

**Figure 1:**
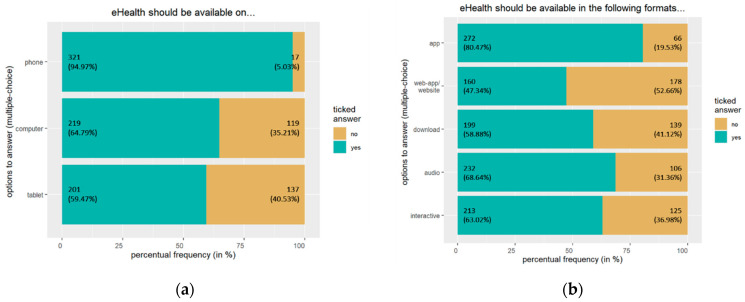

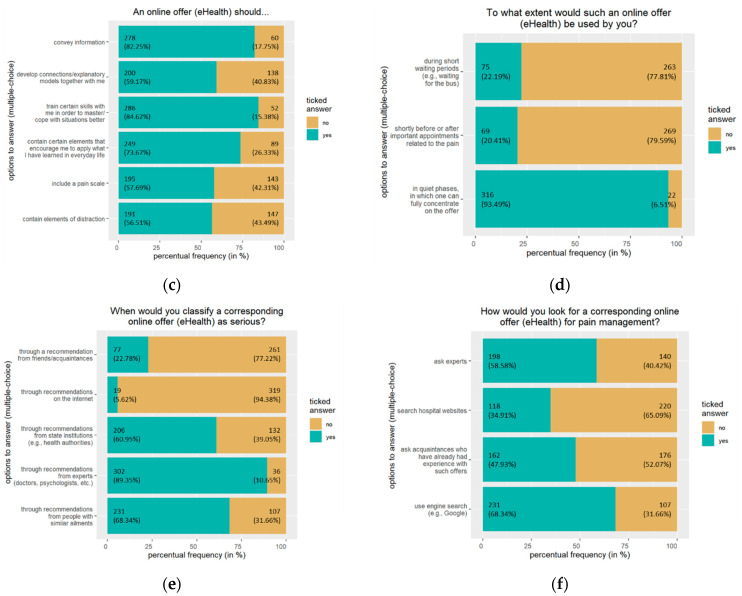
*n* = 338. The bars show absolute frequency (percentual frequency). Shown above are the participants’ responses regarding (**a**) the availability of an online offer for pain management; (**b**) the format of an online offer for pain management; (**c**) the thematic aim of an online offer for pain management; (**d**) their usage of an online offer for pain management; (**e**) the seriousness of recommendations for an online offer; (**f**) their research for an online offer for pain management.

Almost all participants indicated a preference for the availability of an online offer (i.e., eHealth) on the phone, preferably as a mobile app; see Figure 1a,b. In terms of topics, there was a wide range of goals that should be covered by an online offer, with all proposed topics being supported by more than half of the participants; see Figure 1c. Approximately nine out of ten people would want to use an online offer in quiet phases where they would be able to concentrate; see Figure 1d. Expert opinions played a critical role for most participants, both for recommendations from eHealth; see Figure 1e; and for personal research; see Figure 1f.

#### 3.2.4. eHealth: Context of the COVID-19 Pandemic

**Figure 2:**
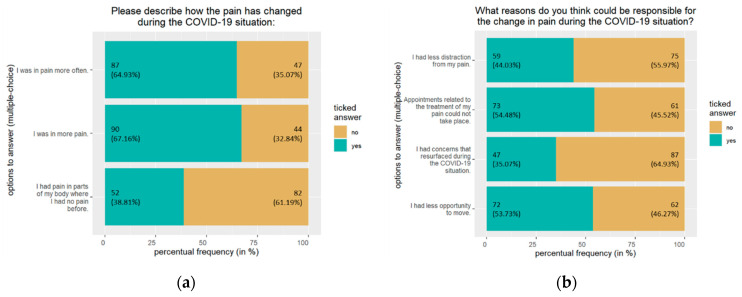
*n* = 134. The bars show absolute frequency (percentual frequency). Shown above are the participants’ responses regarding (**a**) the change in chronic pain during the COVID-19 situation; (**b**) the possible reasons for the change in chronic pain during the COVID-19 situation.

In total, 186 (55.02%) participants stated that their appointments helping with pain management (i.e., doctor visits, therapies, self-help groups, etc.) were canceled due to the COVID-19 situation. Regarding their pain occurrence, 194 (57.39%) participants reported no change during the pandemic situation. However, 145 (42.89%) participants perceived a change in symptoms. While 10 (2.96%) noticed an improvement, 134 (39.94%) participants noticed an aggravation of symptoms. For more information on the participants’ aggravation of symptoms, see Figure 2a. As possible reasons, about half suspected the cancellation of pain-related treatment appointments (54.48%) and reduced possibilities to exercise (53.73%); see Figure 2b.

### 3.3. Cluster Analysis

**Figure 3:**
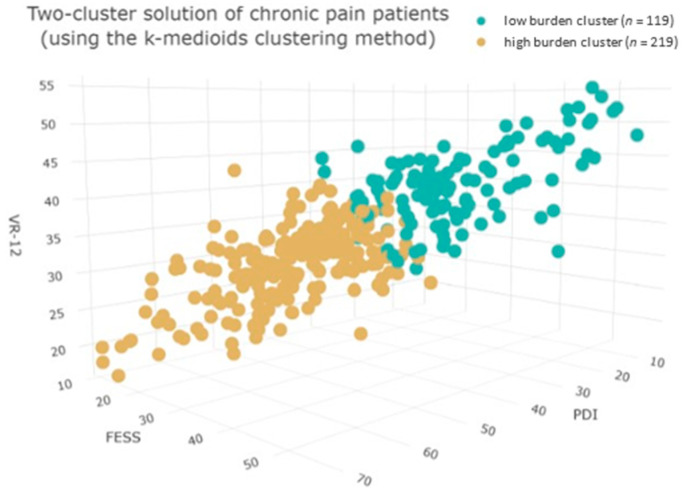
*n* = 338. Shown above is a two-cluster analysis of the *k*-medoids method using the three scales: PDI (perceived pain disability), FESS (pain-specific self-efficacy), and VR-12 (health-related quality of life), in a sample of patients with chronic pain. For the sake of simplicity, the two VR-12 subgroups MCS (mental component score) and PCS (physical component score) were summarized in a VR-12 total mean score.

Using the Hopkins’ *H*-statistic, the data (i.e., PDI, FESS, VR-12) showed a clustering tendency (Hopkins’ *H* = 0.28). Ten out of thirty procedures (33.33%) for determining how many clusters are present returned two clusters. Fewer procedures supported the other nine possible clusters. The *k*-medoids method was used for performing a two-cluster analysis. Model performance of *R*^2^ = 0.375 and an overall classifiability by linear discrimination analysis of 96.45 percent were the overall model’s evaluation results. In detail, a low- (LBC) and high-burden cluster (HBC) could be identified, measured by the mean scores of the mentioned scales. In the LBC, PDI was 28.88, FESS was 39.35, VR-12 MCS was 42.60, and VR-12 PCS 40.03. Contrarily, PDI was 48.14, FESS was 24.95, VR-12 MCS was 33.00, and VR-12 PCS was 27.77 in the HBC. Compared to the LBC, PDI was higher while FESS and VR-12 were lower in the HBC. The two-cluster solution is shown in Figure 3.

#### Group Differences

An examination of the two clusters revealed some significant group differences in the participants’ needs and demands regarding eHealth.

Twenty-five (21.01%) participants in the LBC stated using the internet for research on pain therapists, in the HBC, 93 (42.47%) participants stated the same (*χ*^2^(1) = 15.00, *p* < 0.001, Cramér’s *V* = 0.22). In comparison, the majority of participants in the LBC (66.39%) denied using the internet for the distraction of their pain, and the majority of participants in the HBC (60.73%) agreed to do just that (*χ*^2^(1) = 22.00, *p* < 0.001, Cramér’s *V* = 0.2591).

Compared to participants in the LBC, those in the HBC stated preferring longer support (more than four months vs. permanent) through an online offer for pain management (*p* = 0.001). While most in the LBC (61.34%) disagreed that an online offer for pain management should include distraction elements, most in the HBC (66.21%) agreed that precisely these elements should be included (*χ*^2^(1) = 23.00, *p* < 0.001, Cramér’s *V* = 0.2655).

There was also a significant group difference regarding the change in chronic pain during the COVID-19 situation (*p* = 0.04): while only 31.36 percent in the LBC found a worsening of symptoms, in the HBC, it was 44.29 percent. In both clusters, however, more than half did not perceive any change in their chronic pain (66.95% of the LBC; 52.51% of the HBC). Of the 134 participants who reported symptom aggravation, 30 of 37 (81.08%) in the LBC and 57 of 97 (58.76%) in the HBC stated having symptoms more frequently (*χ*^2^(1) = 4.90, *p* = 0.03, Cramér’s *V* = 0.1317).

## 4. Discussion

Since chronic pain severely restricts the everyday life of those affected [30], having the best possible support system is critical. Since standard medical treatment by, e.g., pain therapists and doctors is usually accompanied by long waiting times [1], an eHealth intervention for pain management may be a resource-friendly supplement that offers immediate and regular support to those affected. This study aimed to determine the needs and demands of those affected by chronic pain in a German sample. In terms of pain duration, frequency, and consequent disability, the sample reflects the high level of burden experienced by people affected by chronic pain. The need for an effective and, above all, available therapy becomes particularly clear in the fact that many patients in the sample reported a perceived ineffectiveness of previous therapies.

Within the sample, optimal conditions for using an eHealth intervention to treat chronic pain were given, with over 99 percent of participants reporting to have private internet access and a long duration of daily use. Nonetheless, two-thirds of the participants stated that they had no experience with eHealth or were unaware of its existence. Similar results were obtained in a cohort of overweight and obese individuals and thus do not appear to relate specifically to the group of people affected by chronic pain [31]. Interventions must, therefore, not only be developed but must also be brought to the attention of potential users. This study primarily reached patients who use digital media in their daily lives, indicating that patients with chronic pain can be effectively reached through social media.

The most frequently used medium, e.g., was clearly the smartphone, which is in line with the findings of Ledel Solem et al. [32], with additional frequent use of PC/laptops or tablets. This may be due to the fact that smartphones, with a number of 63 million in Germany, are widespread and available to the majority of the population [33]. Thus, an intervention should most definitely be available on the smartphone. However, such an intervention could reach even more when available on all devices—such as web applications.

The fact that a large proportion of patients demanded interventions with contact to experts underlines, on the one hand, that the available contact within the framework of the available face-to-face treatments is not enough, but also involves the problem that workforce is still needed. Because the workforce needs to be paid, eHealth would then still fulfill the advantage of location flexibility but not time flexibility and cost-effectiveness [6].

Possible content, such as sharing with other affected individuals, blogs, videos, etc., was rated positively by most participants. They also agreed that an intervention should provide information, opportunities for distraction, and a pain scale, but the desire to learn pain management skills and motivational elements to apply what is learned predominated. This may be an expression of the patients’ desires to actively participate in their treatment. An eHealth pain management intervention could, thus, include several contents to address the needs and demands of the users.

The results showed that an intervention is desired that is permanently available, can be used once a week, and lasts about ten minutes to half an hour per session.

There is a predominant desire to use an intervention mainly in quiet periods, when it is possible to fully concentrate on the intervention, and not on occasion, e.g., in short waiting periods. This shows that eHealth interventions are not only an additional opportunity but are also a serious and promising offer in the eyes of the patients.

Interest in eHealth pain management interventions might be increased if they are viewed as reputable by potential users. This could be achieved primarily by recommendations from experts, which makes it even more important to develop future interventions on a scientific basis [31].

Among chronic pain patients, two groups could be identified reporting either LBC or HBC. As expected, LBC felt less disabled by their pain and felt higher pain-specific self-efficacy and HRQoL than HBC. Older patients seemed to feel a greater burden than younger ones, which could be due to multimorbidity increasing with age [34], or to the fact that the longer one lives, the greater the possibility of suffering from pain for a long time.

Considering the differences in needs and demands between the HBC and LBC offers the opportunity to specifically address patients according to their experienced burden as a further step toward patient-oriented medicine, e.g., LBC patients tended to demand less distraction as the content of an eHealth pain management intervention than patients in the HBC group. This might be due to the higher level or longer history of suffering among HBC patients, which leaves less hope for remission and thus more likely leads to seek distraction rather than a cure. Moreover, less burdened people may use other resources for distraction that require more physical activity, e.g., sports, or may require less distraction due to lower levels of distress. People feeling more burdened stated longer private internet usage per day. The internet does not require much physical activity. Less burdened people, meanwhile, might perform other activities, e.g., exercising [35]. On the other hand, the findings could be explained by experience of pain adversely affecting the performance of tasks that require central attentional control [35]. With increasing intensity of chronic pain, the performance in attention-demanding tasks diminishes [35]. Therefore, chronic pain of LBC might have a lower salience compared to HBC, requiring fewer distractors for an attention shift in a pain management application.

In addition, HBC patients tend to demand longer support than LBC patients. These findings indicate that HBC patients experience such a high level of suffering that they would incur more effort to experience relief. This also raises the question of whether an online offer should have an “expiration date” at all. One option could be a permanent online offer, which can be used as required. In this way, people with various forms of chronic pain [36] could independently dose the duration of the pain management intervention depending on the burden they feel. Maybe in the future, when more data are available, recommendations for a minimum duration of use can be given, depending on the level of the burden.

In line with previous reports by Müßgens et al. [4], we observed that approximately half of those suffering from chronic pain experienced either symptom aggravation or no improvement in their pain during the COVID-19 pandemic. An improvement in symptoms during this period was rather the exception. Similarly, in Canada, socio-economically deprived groups and minorities reported more difficulties in accessing pain relief, health care services, and psychosocial support [37] during the pandemic. Additionally, the pandemic further highlighted the deficiencies in the care of patients with chronic pain. As the effects of the pandemic continue, the pressure to develop alternatives to face-to-face treatment is mounting. Studies to develop science-based treatment options are needed now, as prolonging the pain experienced leads to poorer chances of recovery and worsening of the HrQoL [38].

When interpreting the study results, the following limitations should be considered: Selection bias cannot be ruled out due to online recruitment. It can be assumed that a high proportion of participants have a positive attitude toward digital media and thus toward eHealth offerings. Although the overrepresentation of females is not representative of the gender distribution among patients with chronic pain, chronic pain is reported to be more prevalent in women [39]. The unequal group distribution in sociodemographic, medical, and psychometric data did not allow for an investigation of group differences. According to Goldberg and McGee [30], pain affects all populations but is not distributed equally among the different populations regarding age, sex, income, race/ethnicity, or geography. To obtain more accurate information about the needs and demands of the entire population of patients with chronic pain, a study with a more diverse cohort should be conducted in the future so that the needs of people with chronic pain, e.g., male, older, and less educated, can be addressed. All implications are based on quantitative data being collected in a cross-sectional design (descriptive/correlative). Qualitative surveys and/or longitudinal design could supplement the study results. Furthermore, self-report contains biases (e.g., social desirability, introspection), although self-report is the method of choice for assessing the subjective perception of pain. Potential biases have been minimized by anonymization. Ultimately, self-generated scales/items have been used in addition to validated scales. While this limits comparability, it is due in part to the lack of sufficiently validated questionnaires on eHealth and chronic pain and therefore underlines the need for research in that area. In addition, Cronbach’s alpha was always computed, which indicated adequate internal consistency for all scales.

## 5. Conclusions

To develop an eHealth pain management intervention that is used by those affected by chronic pain, the needs and demands of the users must be addressed to ensure effective therapy in the long term.

If only the most definite results regarding the patients’ needs and demands are summarized, a fairly accurate guide for a future eHealth pain management intervention can already be created. It should be available on the smartphone, permanently available, offer sessions once per week with a duration of 10 to 30 min, and be recommended by experts and offer a variety of content, especially instructions for gaining skills to manage pain and for motivation to utilize them.

## Figures and Tables

**Table 1:** Sociodemographic and psychometric data of participants.

Variable	*n* ^1^	(%) ^2^	*M* ^3^	(*SD*) ^4^
**Gender**				
Male	27	(7.99)		
Female	310	(91.72)		
Diverse	1	(0.00)		
**Educational qualification**				
University degree	75	(22.19)		
High school diploma	90	(26.63)		
Secondary school certificate	131	(38.76)		
Secondary/Elementary school certificate	38	(11.24)		
No school-leaving certificate	0	(0.00)		
Other	4	(1.18)		
**Occupation**				
Retired	49	(14.50)		
Employed	191	(56.51)		
Unemployed	57	(16.86)		
Other	41	(12.13)		
**Place of residence (population size)**				
Large city (>100,000)	96	(28.40)		
Medium sized city (>20,000)	100	(29.59)		
Small town (>5000)	69	(20.41)		
Rural municipality (<5000)	73	(21.60)		
**Relationship status**				
Single	62	(18.34)		
Married	170	(50.30)		
In a relationship	70	(20.71)		
Divorced/separated	32	(9.47)		
Widowed	4	(1.18)		
**FESS ^6^**			30.02	(10.05)
**PDI ^7^**			41.36	(13.05)
**VR-12 ^8^**			34.23	(7.52) ^5^
PCS ^9^			32.08	(9.54)
MCS ^10^			36.38	(11.69)

^1^ Absolute frequencies *h*(*x*). In total, ^2^ Percentual frequencies. In total, ^3^ Mean. ^4^ Standard deviation. ^5^ For the sake of simplicity, the two VR-12 subgroups, MCS and PCS, have been summarized in a VR-12 total mean score. ^6^ Questionnaire for the assessment of pain-specific self-efficacy. ^7^ Pain Disability Index. ^8^ Veterans RAND 12-Item Health Survey. ^9^ Physical component score. In total, ^10^ Mental component score.

## Data Availability

Data supporting the results presented in this article are available upon reasonable request to the corresponding author.

## Appendix B

**Table A1:** Self-generated eHealth items.

Item	Response Option
Do you have private internet access?	Yes/No
How often do you use the following technologies privately? PC/Laptop Tablet/iPad Smartphone/iPhone	Never (I don’t own that)/Rather less (less than 1x/week)/Moderate (more than 1x/week)/Often (daily)/Very often (more than 1x/day)
How long do you use the Internet for private purposes on average per day?	0–1 h/1–2 h/2–3 h/3–4 h/more than 4 h
What do you use the Internet for?	Searching for information about chronic pain/Searching for information about treatment options/Searching for information about pain therapists/News/E-mail/Watching videos/movies/To distract from pain/Social media (Facebook, Instagram, Twitter, etc.)/ Sharing with people in a similar situation/contacting support groups/Search for relaxation exercises/Other (Please name)
Have you already had experience with online offers for pain management (e.g., Pain-T, schmerzApp, Pain Tracer, etc.)? If yes: Which offers have you already used? Please cite.	Yes, I have already made use of online offers for pain management/Yes, I know about the possibility of using online offers for pain management, but I have not tried any yet./No, I am not aware of the possibility of making use of online offers for pain management
If you have already had experience with online offers for pain management: How would you rate your experience with the offerings?	Poor/Rather poor/Neutral/Rather good/Very good
How appropriate do you find the following formats for teaching pain-related content? Personal contact with experts Exchange in groups of those affected in social networks Online offers with expert contact in the context of apps/programs on the Internet Independent support offer as part of an app or as a program on the Internet for the smartphone/iPhone Blogs by those affected/people in a similar situation Exchange in groups of those affected (meeting on site) Online offers with expert contact in social networks Videos/Podcasts Information pages from experts on the internet Independent support offer in the framework of social networks (e.g., Facebook) (Info)emails	Very unsuitable/rather unsuitable/neutral/rather suitable/very suitable
eHealth pain management intervention should be available on:	Smartphone/iPhone/Computer/laptop/Tablet/iPad/Other (Please name)
eHealth should be available in the following formats:	Program on the Internet (web app/website)/Application (app) directly on the device/Information page on the Internet/Material for downloading/Audio/video material (e.g., relaxation exercises set to music)/Interactive tasks and exercises/Other (Please name)
How long should such an online offer for pain management accompany you?	Up to one week/1–4 weeks/1–4 months/Longer than four months/Permanent/Other (Please specify)
In what kind of frequency should the different/respectively new sessions of the online offer be available?	Daily/Weekly/Every two weeks/Other (Please name)
What do you think is an appropriate length for a session in the online pain management service?	1–10 min/10–20 min/20–30 min/More than 30 min/Other (Please specify)
An online offer should…	convey information/work out connections/explanatory models together with me/train certain skills with me in order to better master/manage situations/include certain elements that encourage me to apply what I have learned in everyday life/include a scale to record pain/include elements for distraction/Other (Please name)
To what extent would such an online offer be used by you?	In short waiting periods (e.g., waiting for the bus)/Shortly before or after important appointments that are related to pain/During quiet periods when one can concentrate completely on the offer/Other (Please name)
When would you classify a corresponding online offer as serious?	Through a recommendation from friends/acquaintances/Through recommendations on the Internet/through recommendations from government institutions (e.g., public health department)/Through recommendations from experts (doctors, psychologists, etc.)/Through recommendations from persons with similar complaints/Other (Please name)
How would you look for a corresponding online offer for pain management?	Asking experts/Searching the homepages of hospitals/Asking acquaintances who have already had experience with corresponding offers/Searching in search engines (e.g., Google)/Other (Please name)

*Note*: Items were translated for publication purpose only.
